# Secondhand Smoke Exposure in Public Places and Support for Smoke-Free Laws in Japan: Findings from the 2018 ITC Japan Survey

**DOI:** 10.3390/ijerph17030979

**Published:** 2020-02-04

**Authors:** Genevieve Sansone, Geoffrey T. Fong, Gang Meng, Lorraine V. Craig, Steve S. Xu, Anne C. K. Quah, Janine Ouimet, Yumiko Mochizuki, Itsuro Yoshimi, Takahiro Tabuchi

**Affiliations:** 1 Department of Psychology, University of Waterloo, 200 University Ave W., Waterloo, ON N2L 3G1, Canada; gfong@uwaterloo.ca (G.T.F.); gmeng@uwaterloo.ca (G.M.); lvcraig@uwaterloo.ca (L.V.C.); s4xu@uwaterloo.ca (S.S.X.); ackquah@uwaterloo.ca (A.C.K.Q.); j2ouimet@uwaterloo.ca (J.O.); 2 Ontario Institute for Cancer Research, 661 University Ave Suite 510, Toronto, ON M5G 0A3, Canada; 3 School of Public Health and Health Systems, University of Waterloo, 200 University Ave W., Waterloo, ON N2L 3G1, Canada; 4 Japan Cancer Society, 13th Floor, Yurakucho Center Bldg. 2-5-1, Yurakucho, Chiyoda-ku, Tokyo 100-0006, Japan; mochizuki@jcancer.jp; 5 Division of Tobacco Policy Research, National Cancer Center Japan, 5-1-1 Tsukiji, Chuo-ku, Tokyo 104-0045, Japan; iyoshimi@ncc.go.jp; 6 Cancer Control Center, Osaka International Cancer Institute, Chome-1-69 Otemae, Chuo Ward, Osaka 541-8567, Japan; tabuchitak@gmail.com

**Keywords:** Japan, smoke-free laws, tobacco control policies, smoking, support

## Abstract

Comprehensive smoke-free policies such as those called for by the WHO FCTC are the only way to protect the public effectively from the harms of secondhand smoke (SHS), yet Japan has been slow to implement this important health measure. This study examines baseline levels of smoking and SHS exposure in public places and support for smoking bans in Japan prior to the implementation of the 2018 national smoke-free law. Data are from the International Tobacco Control (ITC) Japan Wave 1 Survey (Feb–Mar 2018), a web survey of adult cigarette smokers, heated tobacco product users, dual users, and non-users (total *N* = 4684). Measures included prevalence of smoking (whether respondents noticed people smoking inside restaurants and bars at their last visit, and workplaces in the last month), and support for complete smoking bans in these venues. Smoking prevalence in each venue was high overall in 2018 (49% of workplaces, 55% of restaurants, and 83% of bars), even higher than in China, the country with the greatest toll of SHS. Support for complete smoking bans was very high overall (81% for workplaces, 78% for restaurants, and 65% for bars). Non-users were less likely to be exposed to SHS and had higher support for smoking bans than tobacco users. These findings point to the ineffectiveness of partial smoke-free laws in Japan and reinforce the call for comprehensive smoke-free laws, which even smokers would support at higher levels than in many other ITC countries.

## 1. Introduction

Secondhand smoke (SHS) is a significant threat to health, estimated to have killed 1.22 million people globally in 2017 [1]. There is no safe level of exposure to SHS. Only comprehensive smoking bans (i.e., those that cover 100% of all indoor public spaces as called for by the World Health Organization Framework Convention on Tobacco Control (WHO FCTC)) are effective in protecting the public from the harms of tobacco smoke [2]. In contrast, partial smoking bans (i.e., those that do not cover all public venues or include allowances for designated smoking areas) are not effective [2,3]. The introduction of national legislative smoking bans is known to lead to improved population health outcomes through reduced exposure to SHS, yet 78% of the world’s population were still not protected by comprehensive smoke-free laws in 2018 [4,5]. The struggle to pass comprehensive smoke-free laws to reduce the massive health threat of SHS continues throughout the world, and this is exemplified in the continuing weak and slow progress toward this important health measure in Japan [6].

Rates of cigarette smoking have declined in Japan in recent years to 18% of adults (30% men, 8% women) in 2015, and are lower than in other Asian countries such as the Republic of Korea and China [7,8]. However, Japan lags behind in the implementation of strong smoke-free laws to protect the public from exposure to tobacco smoke. The 2017 WHO Global Progress Report ranked Japan at the lowest level for smoke-free policy implementation [7]. This lack of smoke-free progress is largely due to the political and social climate in the country, where the tobacco industry has long had a strong presence and influence over government and educational campaigns, contributing to continued social acceptability of smoking [6,9,10,11]. As a result, SHS exposure and its associated harms remain a significant public health challenge. It is estimated that 15,000 non-smokers die each year in Japan from exposure to SHS, and smoking was the leading risk factor for mortality in men in 2015 [12,13].

Japan ratified the WHO FCTC in 2004, but prior to 2018, there was no national smoke-free law. Only two prefectures (Kanagawa in 2010 and Hyogo in 2013) and one city (Bibai in 2016) had implemented their own smoking bans, although these were not comprehensive and lacked strong enforcement [8,9]. Previous legislation under the 2003 Health Promotion Act and Workplace Smoke-free Guidelines encouraged managers of public places and workplaces to take any necessary steps to prevent exposure to SHS, but allowed for designated smoking rooms and did not specify any penalties for failing to restrict smoking [14,15,16].

In light of the upcoming Summer Olympics and Paralympic Games being held in Tokyo from 24 July to 9 August, 2020 (Tokyo 2020), the Health Ministry has taken steps to strengthen smoke-free regulations in Japan. An amendment to the Health Promotion Act banning smoking in all public places was proposed in 2016, but was rejected in 2017 after powerful opposition from the tobacco industry [9]. A weaker version of the law was then passed in July 2018 and will take effect in stages to allow time for compliance, starting 1 July, 2019 and ending with full enforcement by 1 April, 2020 prior to Tokyo 2020 [17]. The amended Act bans all forms of smoking in certain indoor public places, such as schools, hospitals, and government offices starting in July 2019, but allows outdoor smoking areas on the premises (with the condition that necessary measures to prevent SHS exposure among non-smokers must be taken). Indoor smoking will also be banned in restaurants and bars when the law takes full effect, but designated smoking rooms will be allowed, rendering the law to be less than comprehensive. In addition, existing small venues (less than 100 square meters) are currently exempt from the law; thus fewer than half of restaurants and bars will actually be covered by the ban [18,19].

In contrast, the Tokyo Metropolitan Government passed its own smoke-free legislation in 2018, which will take effect in April 2020 and is expected to cover 80% of restaurants [19,20]. Although still not comprehensive, Tokyo’s law is stronger than the national law.

The new regulations of the amended Health Promotion Act have also been extended to include heated tobacco products (HTPs) such as IQOS, glo, and Ploom TECH, which are battery-powered heating devices that heat tobacco rather than burning it, and which have become increasingly popular among smokers in Japan in recent years. Like cigarette smoking, HTP use will be permitted in designated smoking rooms within new or large-scale eating and drinking establishments only accessible to those aged 20 and older and with signs indicating that smoking is allowed. But unlike cigarette smoking rooms, which are to be used exclusively by smokers without eating or drinking, HTPs will be permitted in areas where people are eating and drinking [20,21,22].

Few jurisdictions in Japan have implemented smoke-free regulations, and the few studies evaluating the effectiveness of smoke-free regulations in Japan have focused on workplaces [16,23,24]. For example, survey data from 2011 and 2012 has shown that complete smoking bans in Japanese workplaces were associated with lower prevalence of current smoking, lower levels of self-reported discomfort or ill health due to SHS, and lower SHS exposure compared to workplaces with partial or no bans [23,24].

Given the variation in smoke-free policy implementation across venues, jurisdictions, and types of tobacco products in Japan thus far, and the upcoming changes to smoke-free laws, more research is needed to evaluate existing smoke-free policies in public places and develop evidence-based recommendations for more effective smoke-free laws.

The present study was designed: (1) to measure current prevalence of smoking in public places (workplaces, restaurants, and bars) as reported by large, representative samples of adult cigarette smokers, HTP users, and non-smokers at their last visit, and (2) to assess the level of support among Japanese smokers and non-smokers for comprehensive smoke-free laws. These findings aim to complement recent data comparing the rates of HTP use and cigarette smoking in public places in Japan in 2018 [25], and follows the same design as previous studies evaluating the impact of smoke-free policies in other countries such as China [26]. This study was conducted in 2018, prior to the implementation of the amended Health Promotion Act. We examined differences between respondent type (particularly smokers vs. non-smokers) on both sets of measures, and we compared Japan to other countries—those that have implemented comprehensive laws and those that have partial smoke-free laws. 

## 2. Materials and Methods 

### 2.1. Data Source and Sampling Design

Data are from the International Tobacco Control (ITC) Japan Wave 1 Survey, a web survey of approximately 4500 adults aged 20 and older across 8 geographic regions of the country, who were recruited by email from Rakuten Insight’s Japan web panel. The surveys were conducted from 3 February to 2 March 2018, and closed once the quotas for tobacco users and non-users were achieved. The response rate for the Wave 1 Survey was 45.1%. All respondents gave informed consent before participating in the study, and the survey methods and materials were approved by the University of Waterloo Office of Research Ethics (ORE #31428). The Wave 1 Survey sample was designed to be representative of adult cigarette smokers, HTP users, dual users, and non-users within each region. Table 1 shows the breakdown of the full sample by user type and sample sizes used in this paper. Note that in this study, “smokers” refers to those who smoke cigarettes (either cigarettes only, or dual users of cigarettes and HTPs; excluding HTP only users). Smokeless tobacco product users were not included in the study. For a more detailed description of the ITC Japan Survey methods, see the technical report at the following website: https://itcproject.org/files/JP1-1.5_Technical_Report_28Feb2019_FINAL.pdf/technical-report/.

### 2.2. Measures

The prevalence of smoking and SHS exposure in public places was measured by asking all respondents whether they noticed people smoking inside: (a) restaurants, (b) bars, and (c) workplaces, and results are shown for “yes” responses (Note: the question asked about ‘smoking’ in general, but was in the cigarette smoking section of the survey, so results are interpreted as referring to cigarette smoking.). Specifically, for workplaces, those who worked indoors were asked whether people smoked in their workplace in the last month. For restaurants and bars, those who said they had visited a restaurant/bar in their city in the last six months were asked whether people were smoking inside at their last visit.

Support for smoke-free policies was measured by asking respondents whether they support or oppose a total ban on smoking cigarettes in: (a) restaurants, (b) bars, and (c) workplaces, and (d) whether they support a ban on smoking at the 2020 Tokyo Olympics. For each question, results are shown for responses of “support” and “strongly support” combined (vs. “oppose”, “strongly oppose”, or “don’t know”). 

### 2.3. Data Analysis

All analyses were conducted in SAS with SUDAAN V11 using the multilog procedure (Research Triangle Institute, Research Triangle Park, NC, USA). All results were adjusted for demographic variables (sex and age group). Analytical cross-sectional survey weights were used to account for the complex sampling design for all the analyses to make respondents within each of the user subgroups representative of the corresponding population with respect to demographics and region.

## 3. Results

### 3.1. Smoking Prevalence and SHS Exposure in Public Places

**Figure 1:**
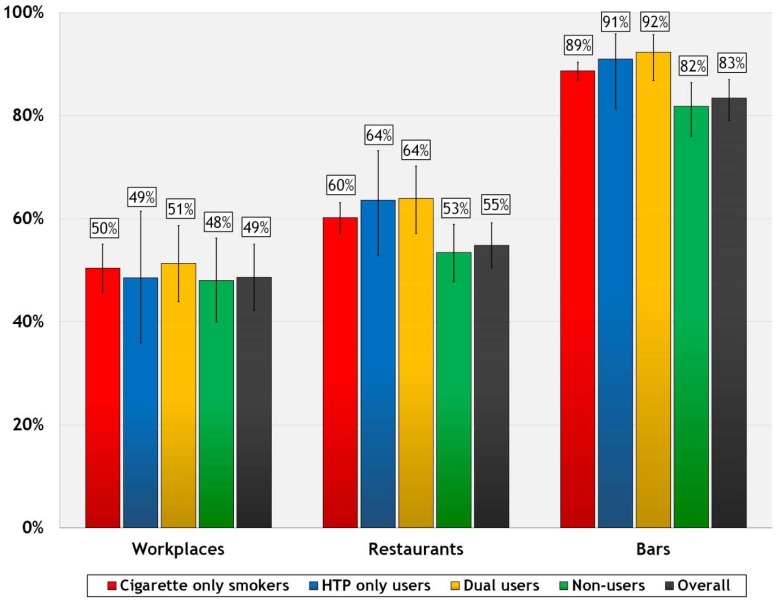
Prevalence of smoking in workplaces, restaurants, and bars, by user group.

As shown in Figure 1, smoking prevalence in public places in Japan was high overall. Almost half of workplaces (49%) had people smoking inside in the last month; over half of restaurants (55%), and 83% of bars had smoking the last time respondents visited these places. Because the cross-sectional weights were calibrated to the population numbers of each of the mutually exclusive user groups, crossed with the 8 geographical regions of Japan, sex, and 4 age groups of the population of Japan aged 20 years and above, these weighted percentages of observed smoking can be taken to be national estimates of smoking prevalence in each of the three venues in Japan.

Results were fairly consistent across user categories, with no significant differences in workplace SHS exposure. For restaurants and bars, cigarette smokers and dual users were each more likely to be exposed than non-users (*p* < 0.05 for each comparison, except for dual vs. non-users in bars, *p* < 0.01). HTP users were also more likely to be exposed in bars than non-users (91% vs. 82%, *p* < 0.05), but not in restaurants.

### 3.2. Support for Smoke-Free Policies

**Figure 2:**
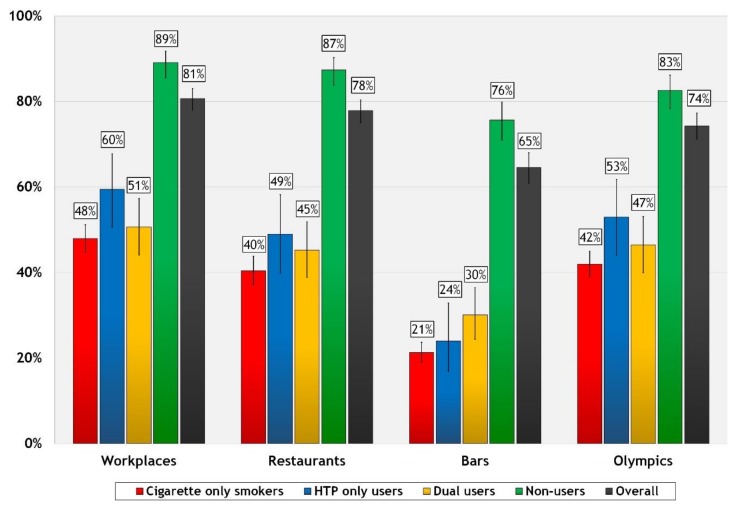
Percentage of respondents who support or strongly support complete smoking bans in workplaces, restaurants, bars, and at Tokyo 2020, by user group.

Overall support for complete smoking bans in public places was high in Japan in 2018—over three-quarters of respondents supported a total ban on smoking cigarettes in restaurants (78%) and workplaces (81%) and two-thirds supported a ban in bars (65%) (see Figure 2). Support for smoke-free Olympics was also high overall (74%). Across the user groups, support was significantly higher among non-users than any other user group, for each venue (*p* < 0.01 for each comparison). Other differences included higher support for smoke-free workplaces and Olympics among HTP users than cigarette smokers (*p* < 0.01 and *p* < 0.05, respectively), and higher support for smoke-free bars among dual than HTP users (30% vs. 24%, *p* < 0.001).

## 4. Discussion

### 4.1. Summary of Findings

This study shows that the lack of strong smoke-free laws in Japan has led to extremely high rates of exposure to deadly SHS in public places. The findings demonstrate not only that Japan needs to implement a comprehensive smoke-free law without exceptions, but also that the Japanese public—including many smokers themselves—are supportive of such a law.

Evidence from Japan and many other countries shows that partial smoking bans are not effective [2,3]; however, previous research has found that the majority of restaurants and bars in Japan in 2018 still allowed smoking in some areas, in accordance with the existing allowances under national legislation [25]. As a result, this study found that smoking and SHS exposure in these venues was very high—especially for bars. Approximately half of all workplaces (49%) and restaurants (55%) and over three-quarters of bars (83%) had smoking in 2018. Another important finding from this study is that overall, the majority of the Japanese public agree that cigarette smoking should be completely banned in public places. As expected, support for complete smoking bans was lowest among cigarette smokers and much higher among non-users, and non-users were also less likely to be exposed to SHS in public places than tobacco users. However, there were few differences between types of tobacco users (cigarettes, HTP, and dual users) on each of the outcome variables.

### 4.2. Comparison with Other Studies

There is little previous data on SHS exposure in Japan, but data from 2010 showed that exposure at work among adults (46% of men and 18% of women) was similar to the current findings [8]. This suggests that there has been no improvement in recent years in SHS exposure in workplaces, owing to the lack of smoke-free laws implemented during this time. Our findings on SHS exposure are also consistent with existing data from the ITC Japan 2018 Survey which shows the high rates of reported cigarette smoking in public places, in comparison to HTP use [25].

Comparisons with other ITC Asian countries with a high burden of tobacco use show the need for stronger global efforts to reduce tobacco use and SHS exposure. ITC cross-country comparisons using identical survey measures of smoke-free policy evaluation suggest that smoking prevalence in public places in Japan in the current study may be as high or higher than in other Asian countries before the implementation of smoke-free laws [27]. For example, the overall prevalence of smoking in restaurants was 55% in Japan in 2018, compared to 59% in China in 2013–2015 (as reported by both smokers and non-smokers in five cities)—where no national smoke-free laws were in place [26]. Smoking was even more prevalent in workplaces and in bars in Japan (49% and 83% overall, respectively) than in China (44% and 79% overall in cities). In contrast, in countries where comprehensive smoking bans in line with WHO FCTC Article 8 have been implemented, smoking prevalence in public places has been dramatically reduced or nearly eliminated. For example, after smoke-free laws in Ireland (2004) and France (2008), smoking in restaurants decreased from over 70% to less than 5%, which was maintained in the long term [28,29,30]. 

ITC Project data also shows that support for smoke-free policies is higher overall in Japan than in other countries. For example, the percentage of Japanese cigarette-only smokers who support smoke-free bars (21%) is higher than the percentage of smokers in Ireland (12%) and France (14%) before those countries implemented their national smoke-free laws [27]. The overall level of public support in Japan is also much higher than the overall level found among smokers and non-smokers in China on the same measures (e.g., 65% of all respondents in Japan vs. 41% of all respondents in Chinese cities support smoke-free bars) [26]. Support for smoke-free laws in 2018 is also greater than the level of support found in earlier studies in Japan. For example, survey data from 2007 showed that only 4% of smokers and 35% of non-smokers supported a complete smoking ban in workplaces; and that the majority of the Japanese public supported partial smoking bans over complete bans [8]. The findings from the current study suggest that support for smoke-free laws may have increased in recent years in Japan, and data from the ITC Project has also shown that public support for smoke-free laws tends to increase even further after they are implemented in a country—especially for comprehensive laws [31,32,33]. 

### 4.3. Strengths and Limitations

This study is the first national evaluation of Japan’s lack of comprehensive smoke-free laws on smoking prevalence in public places and support for such laws among a large, nationally representative sample of cigarette smokers, HTP users, and non-users. While previously published data has also reported on rates of cigarette smoking in indoor public places by smokers themselves [25], this study includes data from both tobacco users and non-users to measure overall national estimates for the prevalence of observed smoking and SHS exposure. This same method for estimating overall prevalence has also been used in other ITC studies where non-users were sampled [26]. However, as this is a baseline cross-sectional study, causal inferences about the impact of smoke-free laws (or the lack thereof) cannot be made. An additional limitation in this study is the use of self-report measures which were not validated and may be subject to recall bias; however, these same measures have been used in many other published studies to evaluate smoke-free policies, including over 50 studies published from ITC Project data across over 20 countries [3,26,29,34,35]. Further research with additional waves of the ITC Japan Survey will be conducted to evaluate the more extensive smoke-free laws that will be implemented in April 2020, including any differences in the impact of laws on cigarette smoking compared to HTP use in public places. 

### 4.4. Implications

While the findings suggest that public support is not a barrier for smoke-free action, the influence of the tobacco industry in Japan remains an obstacle. For example, Japan Tobacco has a long history of promoting “smoking manners” to allow smokers and non-smokers to coexist in the absence of strong smoke-free laws [11]. This strategy has included various media campaigns and sponsoring the establishment of indoor/outdoor smoking areas, despite strong evidence that designated smoking areas fail to protect from the harms of SHS. This strategy is also being used in China—another country where the tobacco industry has a powerful influence over policy-making. Efforts to develop and implement a national smoke-free law in China have halted, with the focus now on the State Tobacco Monopoly Agency’s movement to build a “civilized smoking environment” consisting of indoor smoking rooms across the country [36,37].

Although Japan has made recent progress with the passage of new smoke-free legislation since this study was conducted, evidence has shown that only comprehensive laws, along with strong implementation and enforcement, are effective. But even if a more comprehensive law is implemented, the rising use of HTPs in Japan and the promotion of a “smoke-free” vision by the tobacco industry may increase the challenges of enforcing the law depending on whether HTPs remain subject to the same restrictions. As a Party to the WHO FCTC, Japan should follow the recommendations of the WHO, which has recently decided that HTPs are tobacco products and are therefore subject to the same provisions of the FCTC as cigarettes, including smoke-free policies [38].

## 5. Conclusions

This study provides baseline evidence that the lack of a comprehensive national smoke-free law in Japan prior to 2018 has resulted in an extremely high prevalence of exposure to deadly SHS in indoor public places and workplaces. A comprehensive and strongly enforced law in line with the WHO FCTC Article 8 Guidelines would achieve substantial reductions in SHS exposure and its resulting harms in Japan, as shown by evidence from other ITC countries. 

While new smoke-free legislation will be implemented in Japan by April 2020, that law will still not be comprehensive. The findings of this study demonstrate that the Japanese public would be supportive not only of a smoke-free Olympics, but also of a complete smoking ban covering all indoor public places without exceptions. 

## Figures and Tables

**Table 1:** Study sample.

User Type	Definition	*N*	Percent
Cigarette smokers	Smokes cigarettes at least monthly and uses HTPs less than weekly or not at all	3306	70.6%
HTP users	Uses HTPs at least weekly and smokes cigarettes less than monthly or not at all	207	4.4%
Dual users	Smokes cigarettes at least monthly AND uses HTPs at least weekly	555	11.8%
Non-users	Smokes cigarettes less than monthly or not at all AND uses HTPs less than weekly or not at all	616	13.2%
Total		4684	100%
